# A collaborative study by the Working Group on Hemostasis and Thrombosis of the Italian Society of Clinical Biochemistry and Clinical Molecular Biology (SIBioC) on the interference of haemolysis on five routine blood coagulation tests by evaluation of 269 paired haemolysed/non-haemolysed samples

**DOI:** 10.11613/BM.2018.030711

**Published:** 2018-10-15

**Authors:** Chiara Novelli, Matteo Vidali, Bruno Brando, Benedetto Morelli, Giovanna Andreani, Marina Arini, Paola Calzoni, Roberta Giacomello, Barbara Montaruli, Emanuela Muccini, Angela Papa, Paola Pradella, Lucia Ruocco, Fosca Siviero, Filomena Gemma Viola, Mario Zanchetta, Lorena Zardo, Giuseppe Lippi

**Affiliations:** 1 Transfusion Center and Haematology Laboratory, Western Milan Area Hospital Consortium, Legnano General Hospital, Legnano, Italy; 2 Clinical Chemistry Unit, Maggiore della Carità Hospital, Novara, Italy; 3 Synlab Laboratory, Castenedolo, Italy; 4 Clinical Chemistry Laboratory, Apuan Hospital, Massa, Italy; 5 Clinical and Microbiological Analysis Laboratory, Dell’Angelo Hospital, Mestre, Italy; 6 Clinical Pathology, University Hospital of Siena, Siena, Italy; 7 Department of Medical Area, University of Udine, Department of Laboratory Medicine, ASUI UD, University Hospital, Udine, Italy; 8 Laboratory Analysis, A.O. Ordine Mauriziano, Turin, Italy; 9 Clinical Biochemistry Laboratory, Azienda O.U. S. Giovanni Battista, Turin, Italy; 10 Laboratory Medicine, G. Monasterio Foundation, CNR-Regione Toscana, Pisa, Italy; 11 Transfusion Medicine, University Hospital “Ospedali Riuniti” of Trieste, Trieste, Italy; 12 Clinical Analysis Laboratory, University Hospital of Pisa, Pisa, Italy; 13 Laboratory Medicine, Bassano del Grappa Hospital, Bassano del Grappa, Italy; 14 Department of Laboratory Medicine, Tor Vergata University Hospital of Rome, Rome, Italy; 15 Laboratory Analysis, Degli Infermi Hospital, Ponderano, Italy; 16 Laboratory Analysis, San Giacomo Apostolo Hospital, Castelfranco Veneto, Italy; 17 Section of Clinical Biochemistry, University of Verona, Verona, Italy

**Keywords:** blood coagulation tests, haemolysis, preanalytical phase

## Abstract

**Introduction:**

Haemolysis is the leading cause of sample rejection in laboratory haemostasis. Most studies focused on artificially haemolysed samples. The aim of this study was a prospective assessment of spontaneous haemolysis on haemostasis tests, by comparing results of haemolysed (H) *versus* new, non-haemolysed (NH) specimens, collected within 4hrs. As new coagulometers can identify interfering substances, visual assessment of haemolysis was also compared with instrumental haemolysis index and stratified in subclasses.

**Materials and methods:**

Two hundred and sixty nine paired samples were collected and analysed using ACL TOP750-CTS (Instrumentation Laboratory, Bedford, USA), for prothrombin time (PT), activated partial thromboplastin time (aPTT), D-Dimer (DD), fibrinogen (Fib) and antithrombin (AT). Bias between H and NH was calculated and compared with the respective critical difference (CD).

**Results:**

Mean bias was - 0.1 s for PT (P = 0.057), - 1.1 s for aPTT (P < 0.001), 1025 ng/mL for DD (P < 0.001), - 0.04 g/L for Fib (P = 0.258) and 1.4% for AT (P = 0.013). Bias exceeding the CD varied according to the method, with larger differences for aPTT (36.1%) and DD (17.1%) and < 8% for PT, Fib and AT. No correlation emerged between free haemoglobin values and difference in haemostasis tests in H and NH samples for any tests. Moderate/severe haemolysis involved > 95% of samples. The agreement between visual assessment and instrumental evaluation of haemolysis was 0.62.

**Conclusion:**

Spurious haemolysis deeply influences aPTT and DD, and to a lesser extent AT and Fib. Prothrombin time seems only slightly influenced, suggesting that PT can be accepted also in haemolysed samples. Although a good inter-observer correlation of haemolysis evaluation was found, the instrumental assessment of haemolysis seems recommendable.

## Introduction

The frequency of exclusively analytical errors is generally low in haemostasis testing. However, preanalytical problems are now the leading source of poor testing quality and diagnostic errors. Among the various preanalytical problems, haemolysis is the leading non-conformity, also for samples collected for haemostasis testing. Haemolysis is most frequently due to challenging venipuncture (*i.e.,* often occurring in patients with difficult or fragile veins), excessive vacuum aspiration, prolonged venous stasis, or to the use of too narrow needles (*1*).

Most of the currently available guidelines agree that grossly haemolysed samples should not be tested. The Clinical and Laboratory Standards Institute (CLSI) recommends that all samples with gross haemolysis should be rejected. It is also suggested that those with slight haemolysis can be analysed and test results can then be reported with a note informing that data were generated using slightly haemolysed specimens (*2*). The guidelines of the British Committee for Standards in Haematology (BCSH) also recommend rejecting haemolysed samples, but no distinction is made based on the haemolysis grade (*3*). Even more importantly, both documents failed to provide straightforward thresholds of cell free haemoglobin (fHb) after which haemostasis testing would be analytically and clinically biased. A vague indication is only contained in a separate article, which identified an fHb value of 0.6 g/L as the maximum tolerable cut-off for haemolysis, above which test performance would be biased (*4*).

Many studies have been published on the influence of haemolysis on haemostasis tests (in particular on prothrombin time (PT) and activated partial thromboplastin time (aPTT)), although most of them have been carried out by spiking artificially haemolysed blood to normal plasma, with heterogeneous findings on test sensitivity to haemolysis (*5*-*8*). Therefore, these studies did not reproduce the real world scenario, where haemolysis mostly occurs as a consequence of inappropriate procedures during collection, handling, transportation and storage of blood samples. A limited number of studies is also available on the direct comparison between haemostasis testing performed in paired haemolysed/non-haemolysed samples, but with quite dissonant conclusions (*5*, *6*). Prothrombin time and aPTT seemed affected only in patients but not in healthy subjects, whereas the effect on PT and fibrinogen (Fib) was found not clinically significant (*5*, *6*). These differences may be partially explained by different analytical methods and instruments (*i.e.* photo-optical *vs* electro-mechanical), reagent sensitivity, but also by experimental design (*i.e.* timing of redrawing).

Irrespective of the heterogeneous data published on this important aspect, the failure to identify poor quality plasma specimens may generate a vast array of unfavourable outcomes, including inappropriate clinical decision making based on unreliable data, additional expenses due to sample redraw, and prolonged turnaround time (*9*, *10*). In this regard, new generation haemostasis analysers are equipped with special modules designed for monitoring sample quality and for identifying the most frequent preanalytical problems such as haemolysis, icterus, turbidity/lipemia (HIL), and presence of clots or insufficient sample volume (*8*).

The aim of this prospective collaborative study was to identify the bias in results of the five most frequently ordered haemostasis assays, performed in paired haemolysed (H) and non-haemolysed (NH) plasma samples, collected from the same patient over a short-term period. Moreover, the concordance of visual assessment of haemolysis by two operators and instrumental haemolysis index was evaluated.

## Materials and methods

### Study design

The study was planned by the Working Group on Hemostasis and Thrombosis of the Italian Society of Clinical Biochemistry and Clinical Molecular Biology (SIBioC), and involved 15 separate hospital laboratories allocated in six different Italian Regions. All 277 samples were collected during a 3-month period. All participant laboratories adopted an identical procedure for managing H samples (*i.e.* entailing detailed criteria for identifying haemolysed samples and for timely requesting another specimen). Notably, the procedure used in this study to reject or accept blood coagulation samples was exactly the same used by all participants in their respective routine setting. All samples visually judged as haemolysed were rejected and a new sample was immediately requested and redrawn under the same conditions. Main inclusion criteria of this study were: (i) availability of a second NH plasma sample within 4 hours from collection, and (ii) fulfilment of all the main preanalytical requirements, such as the collection in the appropriate blood tube (*i.e.,* evacuated blood tube containing 3.2% sodium citrate, appropriate blood to anticoagulant ratio, lack of visible clots). To exclude additional confounding factors other than haemolysis, samples judged at the collection site as lipemic and/or hyperbilirubinemic were excluded from the study.

Information on anticoagulant therapy and hospital department was collected whenever available to the laboratory. All tests were performed on residual plasma after the ordered tests had been completed; therefore, no patient informed consent was necessary. The study was approved by the local scientific Committees of all centers where samples were collected.

### Blood collection and storage

In each center, blood samples were collected into evacuated non-paediatric blood tubes containing 3.2% (109 mmol/L) buffered sodium citrate, with a 1:9 ratio between anticoagulant and blood (each hospital used the locally available tube type and brand). After visual inspection for assessing accuracy of blood to anticoagulant ratio and lack of visible clots, the blood tubes were immediately centrifuged at 1500xg for 15 minutes at room temperature. The presence of haemolysis in centrifuged plasma was visually assessed by the laboratory staff and, when the samples were considered H, a second sample was immediately requested. The second sample was then managed exactly as the former. Two aliquots of 0.4 mL of plasma of both samples (*i.e.,* H and NH) were immediately stored in cryotubes at - 80 °C in each center. All plasma aliquots were made anonymous and labelled with (i) a serial number, (ii) a letter identifying the local hospital, and (iii) a code identifying haemolysed (*i.e.,* “H”) and non-haemolysed (*i.e.,* “NH”) aliquots. The maximum storage time of frozen plasma samples before delivery was 6 months. All sample batches were transported under controlled temperature in dry ice (not higher than - 40 °C) on the same day from all centers to the reference laboratory, where analyses were performed (Legnano General Hospital, Legnano, Milan, Italy). Upon receipt samples were stored again at - 80 °C. Twelve days passed between the receipt of all batches and the processing of the last sample in the last analytical session, always keeping the storage temperature at - 80 °C.

### Laboratory testing

Haemolysis influence was studied on the paired results of the five most commonly performed laboratory tests: PT, aPTT, Fib, Dimer-D (DD) and antithrombin (AT). All tests and automatic haemolysis assessment were performed using the same instrument (ACL TOP 750-CTS, Instrumentation Laboratory, Bedford, USA) and using proprietary reagents (Instrumentation Laboratory, Bedford, USA) as follows: PT, HemosIL RecombiPlasTin 2G (reference range: 9.1 - 13.7 sec, ratio 0.80 - 1.20, ISI = 1.00); aPTT, HemosIL SynthASil (reference range: 24.6 - 37.0 sec, ratio 0.80 - 1.20); D-Dimer, HemosIL HS-DD (reference range < 270 ng/mL); Fib, HemosIL QFA Thrombin (reference range: 1.8 - 4.0 g/L) and AT, HemosIL Liquid Antithrombin (reference range: 80 - 120%). The same lot of reagents was used for all analytical sessions. All samples were thawed at 37 °C for 5 min in a water bath immediately before testing.

The tests were completed in 12 different analytical sessions of 20 - 25 paired plasma samples over five consecutive days, using a repetitive H/NH sample sequence. Internal quality controls (HemosiL Normal and Abnormal Control, Instrumentation Laboratory, Bedford, USA), were performed at the beginning of each session and every 2 hours.

### Haemolysis assessment

After the delivery of all frozen aliquots to the reference laboratory, the thawed plasma, previously evaluated at the collection site, was visually re-evaluated by two independent laboratory technicians. The sample hue was compared with a predefined photographic colour scale in 9 ranks, on the basis of predefined quantities of free haemoglobin (fHb) added to clear plasma, as published by Lippi *et al.* (*11*). In the attempt to correlate the visual colour scale to an instrumental measure, the haemolysis degree was ranked in 9 different classes, similar to Lippi et al. (Table 1), from no haemolysis (*i.e.,* ≤ 0.05 g/L of fHb) up to gross haemolysis (*i.e.* > 10 g/L of fHb) (*4*). All aliquots were then loaded in the ACL TOP 750-CTS analyser and a spectrophotometric preanalytical assessment of HIL was performed, with the intent of correlating the subjective evaluation of plasma hues into more objective instrumental measurements. Briefly, instrumental haemolysis assessment is based on optical absorbance measurement of diluted samples at three different wavelengths (671 nm for lipid-related turbidity; 535 nm for turbidity and fHb; 405 nm for fHb and bilirubin) (*8*). An automatic algorithm is then used to resolve mathematically the crude readings and to generate a semi-quantitative measure (*i.e.,* g/L for haemoglobin, µmol/L for bilirubin and milli-Absorbance units (mAbs) for turbidity) of the three HIL indices (*8*).

**Table 1:** Classification of haemolysis based on free haemoglobin values in plasma samples and visual inspection

**fHb**	**Haemolysis degree**	**Plasma colour hue**	**Haemolysis rank**
≤ 0.05	Non-haemolysed	yellow	1
0.05 - 0.30	Slightly haemolysed	yellow to slightly pink	2 - 3
0.31 - 0.60	Mildly haemolysed	pink to slightly red	4
0.61 - 2.00	Frankly haemolysed	slightly red	5
2.00 - 5.00 5.01 - 10.00 > 10.00	Grossly haemolysed	red to brown	6 7 8 - 9
fHb - concentration of free haemoglobin (g/L).			

### Statistical analysis

Statistical analyses were performed by SPSS statistical software v.15.0 (SPSS Inc., Chicago, USA). Qualitative variables were summarized as absolute numbers or relative frequencies, whilst quantitative continuous variables were reported as median, interquartile range (IQR) and minimum-maximum, respectively. According to q-q plot, Kolmogorov–Smirnov and Shapiro–Wilk tests, data were found to be not normally distributed. Differences between H and NH samples were evaluated by non-parametric paired sample test and Bland-Altman analysis. The 95% interval of differences between H and NH samples were assessed as 2.5^th^ and 97.5^th^ percentiles. The correlation between fHb concentration of H samples and H-NH differences was evaluated for each coagulation test with non-parametric Spearman’s test. Clinically significant or critical differences (CD) between H and NH samples for each test were defined using the formula described by Jones *et al*.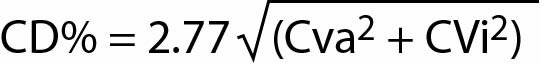 (*12*, *13*). The formula considers the analytical variability (CVa), the intra-individual biological variation (CVi) and the fact that the CD includes variation in two measurements (where:
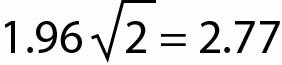
). Table 2 shows the between-run CVa calculated centrally using control materials and the CVi, as reported by Fraser *et al*. (*14*). Inter-operator agreement and agreement between visual inspection and HIL was assessed by Cohen’s kappa with linear weighting after transformation of HIL data in ordinal variables (Table 1). Moreover, the values of PT ratio were transformed into four categorical - although empirical - variables, according to established therapeutic PT ranges: normal range (0.80 - 1.20); anticoagulation below therapeutic range (1.21 - 2.00); anticoagulant in therapeutic range for most patients taking vitamin K antagonists (2.01 - 3.50); and anticoagulant over therapeutic range (> 3.50).

**Table 2:** Critical differences for PT, aPTT, DD, Fib and AT applied in this study

**Coagulation test**	**CVi (%)**	**CVa (%)**	**CD (%)**
PT	4.0	1.3	11.7
aPTT	2.7	1.5	8.6
DD	23.3	2.5	65.0
Fib	10.7	6.0	34.0
AT	5.2	2.5	16.0
aPTT - activated partial thromboplastin time. AT - antithrombin. DD - Dimer-D. Fib - fibrinogen. PT - prothrombin time. CD - critical difference. CVi - intra-individual biological variation according to (14). CVa - between-run analytical variability, as calculated by the central laboratory.			

In order to evaluate the analytical variability for all methods, following the Clinical Laboratory Standards Institute (CLSI) indications, both normal and abnormal quality control materials were measured 20 times/day for 5 days (*15*). Imprecisions (both within- and between-run coefficient of variation; CV%) were calculated as standard deviation/mean ratio.

## Results

Overall, 277 paired samples were originally collected according to our inclusion criteria and shipped to the reference center. Three of these samples ought to be excluded due to high concentration of other interfering substances (*i.e.,* triglycerides > 16.4 mmol/L and/or bilirubin > 232.6 µmol/L) (*8*). Five additional samples, which had been originally classified as NH in the collecting center, were also excluded since they were then reclassified as H by the reference center. Therefore, the final sample size included 269 specimens. The samples were collected in the following wards: emergency department (57%), internal medicine (21%), surgery (9%), intensive care unit (7.3%) and outpatient clinic (5.7%). The origin of samples did not influence the study results (data not shown).

The values of PT, aPTT, DD, Fib and AT measured in both H and NH samples are shown in Table 3. The median value of fHb measured on ACL TOP 750-CTS was 1.96 g/L (IQR, 1.29 - 2.76 g/L) and 0.29 g/L (IQR, 0.29 - 0.29) in H and NH aliquots, respectively. Bland-Altman plot analysis between H and NH aliquots yielded the following results (mean bias, 95% CI for bias, 95% of the differences H-NH as 2.5-97.5^th^ percentiles): - 0.1 s (- 0.6 to 0.4 s and - 3.7 to 4.3 s) for PT, - 1.1 s (- 1.8 to - 0.3 s, and - 12.3 to 8.4 s) for aPTT, 1025 ng/mL (366 to 1683 ng/mL and - 558 to 13,023 ng/mL) for DD, - 0.04 g/L (- 0.11 to 0.03 g/L and - 1.42 to 1.27 g/L) for Fib, and 1.4% (0.3% to 2.5% and - 13% to 18%) for AT. No significant correlation was observed between fHb in haemolysed samples and the differences of haemostasis test results between H and NH aliquots (r = 0.16 for PT; r = 0.18 for aPTT; r = - 0.05 for DD; r = 0.10 for Fib; r = 0.04 for AT; P ranging from 0.051 to 0.519). The percentage of samples with differences of haemostasis test results between H and NH aliquots (H-NH) exceeding the CD are shown in Table 4. The figures further decrease when only the values which could lead to different clinical interpretations are taken into account, as shown in Table 4. In other words, results of H and NH samples falling within the normal reference range and resulting in no clinical actions were excluded. The comparison of PT values obtained in H and NH aliquots after transformation into arbitrary categorical variables is shown in Table 5. Overall, the number of concordant pairs was 247 (91.8%), with a Cohen’s kappa of 0.90 (95% CI, 0.86 - 0.94). A substantial difference could only be appreciated in four paired samples, which differed for more than 1 class (*i.e* the PT result of the H sample fell in the > 3.50 class, and the respective NH sample was in the 1.20 - 2.00 class).

**Table 3:** Coagulation tests results investigated in paired haemolysed and non-haemolysed plasma aliquots

**Assay**	**Haemolysed samples** **(N = 269)**		**Non-haemolysed samples** **(N = 269)**		**P**
	**median (IQR)**	**min-max**	**median (IQR)**	**min-max**
**PT (s)**	12.9 (11.4 - 21.3)	9.6 - 246.9	13.0 (11.4 - 21.9)	9.9 - 286.8	0.057
**PT (ratio)**	1.13 (1.00 - 1.87)	0.84 - 21.77	1.14 (1.00 - 1.92)	0.87 - 25.16	0.056
**aPTT (s)**	31.8 (28.4 - 37.4)	19.2 - 77.2	33.6 (29.5 - 37.6)	22.0 - 80.6	< 0.001
**aPTT (ratio)**	1.03 (0.92 - 1.21)	0.62 - 2.51	1.09 (0.96 - 1.22)	0.71 - 2.62	< 0.001
**DD (ng/mL)**	392 (203 - 923)	25 - 59,713	317 (173 - 668)	36 - 28,695	< 0.001
**Fib (g/L)**	3.76 (3.04 - 5.06)	0.97 - 10.98	3.89 (3.06 - 5.37)	1.15 - 10.50	0.258
**AT (%)**	103 (88 - 116)	25 - 153	102 (87 - 115)	26 - 186	0.013
Data are shown as median, interquartile range (IQR) and minimum-maximum. aPTT - activated partial thromboplastin time. AT - antithrombin; DD - Dimer-D. Fib - fibrinogen. PT - prothrombin time. P-values have been obtained with medians of values. P < 0.05 was considered statistically different.					

**Table 4:** Coagulation tests results displaying bias exceeding the critical difference

**Assay**	**Total samples exceeding CD, N (%)** **(N = 269)**	**Samples with results outside the reference range exceeding CD, N (%)** **(N = 126)**

**PT**	20 (7.4)	17 (6.3)
**aPTT**	97 (36.1)^*^	55 (20.4)^*^
**DD**	46 (17.1)	40 (14.9)
**Fib**	11 (4.1)^†^	4 (1.5)^†^
**AT**	17 (6.3)	10 (3.7)
The first column shows the differences beyond CD calculated for the total group of patients. The second column shows the differences beyond CD calculated for 126 samples with H and/or NH values outside the normal reference ranges (percent values were overall lower than in the whole patient group). aPTT - activated partial thromboplastin time. AT - antithrombin. CD - critical difference. DD - Dimer-D. Fib - fibrinogen. PT - prothrombin time. ^*^ highest difference beyond CD. ^† ^lowest difference beyond CD.		

**Table 5:** Agreement between prothrombin time results divided in subclasses obtained in haemolysed and non-haemolysed samples

**PT ratio H subclasses**	**PT ratio NH subclasses**				**Total H samples**
	**0.80 - 1.20**	**1.21 - 2.00**	**2.01 - 3.50**	**> 3.50**
0.80 - 1.20	141	8	0	0	149
1.21 - 2.00	4	52	0	1^*^	57
2.01 - 3.50	0	3	34	2	39
> 3.50	0	3*	1	20	24
**Total NH samples**	145	66	35	23	269
^*^More than one class disagreement. A disagreement of more than one class between H and NH samples may affect major clinical decisions. H - haemolysed aliquot. NH - non-haemolysed aliquot. PT - prothrombin time.					

**Figure 1:**
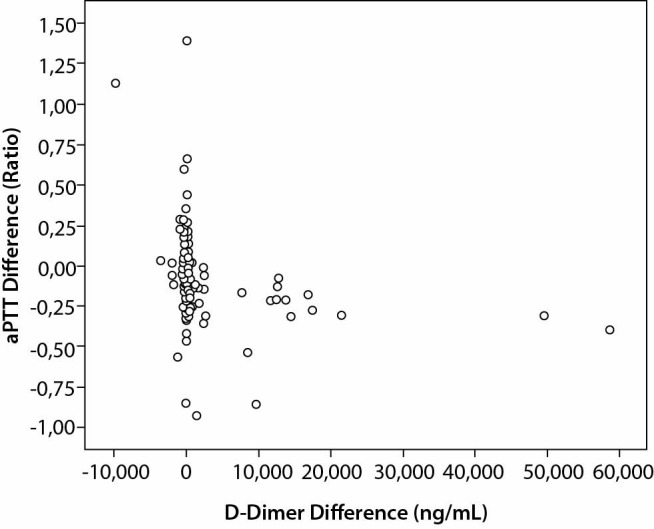
Differences of results of H versus NH samples for D-Dimer and aPTT. For very large D-Dimer differences (> 3000 ng/mL, occurring in 14 paired samples), a trend toward the reduction of aPTT indicates the activation of the coagulation cascade (*17*). aPTT - activated partial thromboplastin time.

The difference between H and NH samples for DD and aPTT is summarized in Figure 1. Interestingly, aliquots with a difference of DD values obtained in H and NH > 3000 ng/mL displayed a significantly lower median difference of aPTT values obtained in H and NH (- 7.6 *vs.* - 0.4 s; P < 0.001) and a lower median difference of Fib values obtained in H and NH (- 0.97 *vs.* 0.00 g/L; P < 0.001), whilst no association was found with difference of PT values obtained in H and NH (- 0.5 *vs*. 0.00 s; P = 0.131) or with difference of AT values obtained in H and NH (- 0.14 *vs*. - 1.00%; P = 0.955).

The overall agreement for ranking haemolysis according to the visual scale was 0.85 (95% CI, 0.81 - 0.89) between the two laboratory technicians, whilst the individual agreement of haemolysis ranking according to the visual scale and HIL data was 0.62 (95% CI, 0.57 - 0.67) and 0.65 (95% CI, 0.60 - 0.70) for each of the two laboratory technicians, respectively (Table 6).

**Table 6:** Haemolysed samples divided in subclasses according to visual inspection and automated assessment

**Haemolysis rank**	**Supernatant haemoglobin (g/L)**	**Automated assessment, N (%)**	**Operator 1, ** **N (%)**	**Operator 2, N (%)**
1	≤ 0.05	0 (0)	0 (0)	0 (0)
2 - 3	0.05 - 0.30	0 (0)	9 (3.4)	10 (3.7)
4	0.31 - 0.60	5 (1.9)	60 (22.3)	50 (18.6)
5	0.61 - 2.00	137 (50.9)	103 (38.3)	106 (39.4)
6 7 8 - 9	> 2.00 2.01 - 5.00 5.01 - 10.00 > 10.01	127 (47.2) 96 (35.7) 24 (8.9) 7 (2.6)	97 (36.0) 51 (19.0) 21 (7.8) 25 (9.2)	103 (38.3) 54 (20.1) 24 (8.9) 25 (9.3)
The number of cases within each category (as classified by automated assessment and by each operator) is reported as number and percentage.				

## Discussion

The results of our study showed that PT is scarcely influenced by spurious haemolysis, whereas a significantly higher bias is observed in the paired comparisons of H and NH samples for aPTT and DD, as described in earlier studies using different analytical platforms (*6*, *8*, *16*). Moreover, when present, the bias was not directly proportional to the degree of haemolysis, probably due the occurrence of other factors, such as the activation of the coagulation cascade. More specifically, a systematic bias, according to Bland-Altman analysis, was not significant for PT and Fib, but was significant for aPTT, DD and to a lesser extent for AT. The percentage of values with bias exceeding the CD was relatively low for PT, Fib and AT, whereas a much higher percentage exceeded the CD for both aPTT and DD. The figures further decrease when the results that could lead to a different clinical interpretation are taken into account, namely only those H and/or NH values that are outside the normal value range, as shown in Table 4. Which percentage of samples with a bias higher than the CD should be considered as significant? From a statistical point of view the cut-off value is usually 5%. However, for large data files with tightly similar values, even small drifts may generate ‘statistically different’ results, as it happened for PT (*i.e.* 7.4% *vs*. 6.3%).

The evidence that PT is seemingly less vulnerable to haemolysis is reinforced by the high concordance observed when PT values are divided in subclasses, with 22 samples being attributed to a different class when measured in H and NH aliquots (Cohen’s kappa = 0.90). Unlike PT, aPTT and DD results were instead markedly biased in H aliquots. In some samples a very high difference between H and NH samples was noted for DD (Figure 1): in such cases an association was observed between the increase of DD and the reduction of aPTT and Fib without changes occurring to PT and AT, thus suggesting the activation of the coagulation cascade. In the present study only one instrument type and reagent lot were used for all analyses, so the influence of many methodological variables can be reasonably considered as minimal.

Notably, the evidence that aPTT may be more influenced than other tests by preanalytical issues is not new, and this can be rather easily explained. Unlike PT, in which the high concentration of thromboplastin in the test reagent is likely to overcome many potential preanalytical drawbacks, the reagents used for aPTT (*e.g.*, micronized silica, as in the current investigation) are extremely sensitive to even mild alterations of blood coagulation occurring throughout blood collection and ultimately mirrored by the presence of haemolysis in plasma (*17*). The lack of association between the haemolysis level and the bias of haemostasis test results, as assessed by very low and not significant correlation coefficients makes it impossible to use correction formulas to adjust data generated in haemolysed samples, as suggested for potassium (*18*). Moreover, the use of correction formulae is generally not widely accepted in blood coagulation tests.

Spurious haemolysis is a substantial issue in laboratory diagnostics, accounting for up to 40 - 70% of all samples rejected for non-conformity. Besides rare cases of haemolytic anaemia (*i.e.*, *in vivo* haemolysis), spurious haemolysis usually reflects a variety of problems occurred during blood collection or immediately afterward (*1*). This issue causes diagnostic delays and also poses considerable economical and organizational burden to the clinical laboratories (*9*, *10*). On the other hand, reporting data for highly haemolysis-sensitive analytes is a questionable practice, since clinical decisions based on unreliable data may jeopardize patient safety (*19*-*21*). The most suitable approach for dealing with haemolysed samples is the request of another specimen, although practices and standard operating procedures are quite heterogeneous among different laboratories. This cautionary practice is driven by data published in many previous articles, which showed that artificially generated haemolysis using various physical or chemical means may have a strong impact on haemostasis testing, depending also on instrument and reagent used (*6*, *16*, *20*-*24*). Interestingly, within this experimental model (haemoglobin-spiked or physically haemolysed samples) even when the same ACL TOP instrument was used, haemolysis was found to interfere at a very different extent on coagulation tests (*8*, *23*).

Our original collaborative investigation was based on a real life scenario, according to which the influence of haemolysis on some haemostasis tests was assessed by analysing paired plasma samples collected from the same patient, within 4 hours, and thus within a time frame in most cases not likely be associated with dramatic changes of patients’ condition. Our approach differs remarkably from previous studies that used artificially haemolysed blood samples (*7*, *16*, *22*). The present study design seems more suited to identify the potential biological influence of haemolysis in routine practice, and also includes a heterogeneous set of samples, collected in separate wards from different categories of patients, either undergoing anticoagulant therapy, or not. To the best of our knowledge, the design of this study also allowed to include the largest sample size ever used for this purpose, whereas the centralization of testing in only one reference laboratory avoided the variability of data obtained in many different centers. Finally, the haemolysis level was assessed directly by spectrophotometric measurement, thus generating data that closely mirror the haemoglobin concentrations measurable with reference cyan-methaemoglobin assays.

Although detailed and common procedures were used by all participant laboratories, we cannot rule out that at least some H-NH differences could be explained by not reported ongoing therapeutic treatments other than oral anticoagulants (*i.e.* intravenous fluids) between samplings or by a rapid change of clinical conditions.

Another important finding emerging from our study is that the visual assessment of plasma quality should be definitively abandoned. Although the inter-operator agreement in ranking the haemolysis level was quite satisfactory (*i.e.* 0.85), the overall agreement of the two laboratory technicians with automatic HIL assessment on ACL TOP 750-CTS was unsatisfactory (*i.e.* 0.62 and 0.65). Therefore, the widespread use of automatic HIL assessment should be further fostered also for haemostasis testing, especially because the vast majority of new haemostasis analysers are now equipped with modules for this rapid and efficient sample quality check.

In conclusion, the results of our study show that a large unfavourable impact of spurious haemolysis was observed for aPTT, DD, and to a lesser extent for AT and Fib. On the contrary, PT seems only slightly influenced by spurious haemolysis, suggesting that this test can be taken as reliable also in haemolysed plasma samples. A good inter-observer correlation of haemolysis assessment was found, but the usage of an instrumental evaluation method seems today more recommendable.
